# Whether all obese subjects both in metabolic groups and non-metabolic groups should be treated or not

**DOI:** 10.1186/2251-6581-13-21

**Published:** 2014-01-29

**Authors:** Moloud Payab, Shirin Hasani-Ranjbar, Bagher Larijani

**Affiliations:** 1 Obesity and Eating Habits Research Center, Endocrinology and Metabolism Molecular - Cellular Sciences Institute, Tehran University of Medical Sciences, Tehran, Iran; 2 Endocrinology and Metabolism Research Center, Endocrinology and Metabolism Clinical Sciences Institute, Tehran University of Medical Sciences, Tehran, Iran; 3 EMRI (Endocrinology and Metabolism Research Institute), 5th Floor, Shariati Hospital, North Kargar Ave., Tehran 14114, Iran

**Keywords:** Obesity, Metabolic disorder, Osteoarthritis, Sleep disorders

## Abstract

More recent researches have focused on metabolically healthy obese (MHO) phenotypes and on this phenotype, individuals may be obese without metabolic disorders. Osteoarthritis (OA), kidney diseases and sleep disorders are three factors related to the obesity that these conditions are associated only with obesity but not with metabolic complications. Regardless of whether obese individuals are in metabolic groups or not, they should be treated. All studies should be based on the risk of all-cause mortality in the MHO phenotypes.

## 

The prevalence of obesity is increasing worldwide and general and abdominal obesity are the major public health and social problem [1]. The most important consequences of obesity include type 2 diabetes, hypertension, hyperlipidemia, coronary heart diseases, ischemic stroke, certain kinds of cancer, osteoarthritis, kidney diseases and sleep disorders [1]. Some of these complications are related to metabolic abnormality associated with obesity and others are related to the obesity directly.

In some studies, association between weight status and metabolic health has been proven but it is not applicable for all subjects [2].

Osteoarthritis (OA), sleep disorders and kidney disease are three complications related to the obesity that must be considered apart from metabolic syndrome for obese individuals. These three conditions are associated with only obesity and over weight but not necessirily with their metabolic complications.

It is established that obesity has been an independent risk factor for osteoarthritis of the hip and knee [3]. It is estimated that knee osteoarthritis will be doubled by 2050 and through decreasing only 1% of BMI, the number of patients with knee osteoarthritis will be reduced by 2050 [4].

And also sleep deprivation is associated with an increase in body weight. Obstructive sleep apnea, insomnia and restless legs syndrome are three of the most common sleep disorders. Several studies have shown that there is a significant association between sleep disorders and obesity [5].

Three phenotypes are known as “metabolically healthy obese (MHO)”, “metabolically nonhealthy nonobese (MNHNO)”, “metabolically nonhealthy obese (MNHO)” [6]. In this classification BMI (Body Mass Index) is the indication of definition.

More recent researches have focused on metabolically healthy obese (MHO) phenotypes [7-9]. On this phenotype, individuals may be obese but metabolic disorders do not exist (e.g. dyslipidemia, insulin resistance, hypertension) [10]. A recent longitudinal study showed that the MHO phenotype is associated with favorable cardiovascular outcomes, but this status is transient at one third of the subjects [11]. Some studies have shown that the subjects with MHO phenotype are not at increased risk of morbidity and mortality and consequently treatment of obesity for this group is unnecessary, but guidelines for treatment of obesity in the United States suggest that regardless of cardiovascular diseases, obese patients should be treated [12]. On the other hand Hamer et al. in their study concluded that individuals with MHO phenotype, were not at increased risk of cardiovascular diseases and mortality risk [13].

In summary, it is established that obese individuals with metabolic problems are at higher risk of mortality than their non-obese counterparts and should be treated in order to decrease cardiovascular events. For metabolically healthy obese individuals there is a gray zone. We recommend that future studies consider the risk of all-cause mortality and also morbidity (certain kinds of cancer, osteoarthritis, kidney diseases and sleep disorders) in the (MHO) phenotypes. This would open a new approach for treatment of all obesity phenotypes.

## Abbreviations

MHO: Metabolically healthy obese; MNHNO: Metabolically nonhealthy nonobese; MNHO: Metabolically nonhealthy obese; BMI: Body mass index.

## Competing interests

The authors declare that they have no competing interests.

## Authors’ contribution

SHR participated in the study design, and given final approval of the version to be published. MP wrote the first draft. BL participated in critical review. Authors extensively edited it and approved the final manuscript.

## Authors’ information

1. Shirin Hasani-Ranjbar: Assistant Professor, Endocrinology and metabolism, Tehran University of Medical Sciences, Iran

2. Moloud Payab: Master of Health Science in Nutrition.

3. Bagher Larijani: Professor of Endocrinology and metabolism, Tehran University of Medical Sciences, Tehran, Iran
